# Supplementary material for: scifi-ATAC-seq: massive-scale single-cell chromatin accessibility sequencing using combinatorial fluidic indexing
Source: Genome Biol. 2024 Apr 8;25:90. doi: 10.1186/s13059-024-03235-5 (PMC11003106; doi:10.1186/s13059-024-03235-5)
Supplement: Supplementary file 3 — Additional file 3. [file 13059_2024_3235_MOESM3_ESM.docx]

Review History

**First round of review**

**Reviewer 1**

**Are you able to assess all statistics in the manuscript, including the appropriateness of statistical tests used?** Yes, and I have assessed the statistics in my report.

**Comments to author:**

This manuscript describes very clearly an elegant high throughput method for single nuclei ATAC-seq. While the method was already published in animals, this represents an important novelty in the field of plant science with numerous applications and a broad audience.

The manuscript is well written, clearly presented, despites a few improvements that can be made, and results support the conclusions.

Major comments:

While this is a short report, more insights on the underlying biology would really improve the quality and value of the manuscript.

One can imagine that a more thorough biological analysis of the generated data will follow this paper in the future, but more attention to the biological relevance of the data produced is needed. For example, did the authors find that maize genetic divergence of the different genotypes is correlated with more differential ACR etc.

Or, for example, does the tropical variety have more or less ACR in specific types of genes?

Is there any GO term enrichment for the differential ACR across genotypes and then which cell type seems to be the most divergent between genotypes etc?

Could the authors also comment on the minimum number of SNPs per genotype to be used here. As typically each read is about R1:49bp and R2 50bp, could you state how many SNPs per kb are necessary to be certain to correctly assign reads to a specific genotype. Maybe add in the manuscript the SNP rate between genotypes.

Fig. 1, k,i

The comparison of data quality between 16k and scifi 100k 200k is biased due to the number of nuclei. It is quite expected to have a better coverage and cell calling with 10 to 20x more nuclei.

Moreover, in L120-121 the resolution of ACR can be difficult to discern as the 100-200k cells are driving the integration and 16k libraries that could have a different batch effect and their clustering can be affected by the common integration. An integration of only the 16k libraires should help the comparison. Do you find the same cell type with only the 16k nuclei, or do you need more nuclei in general?

To perform a more rigorous approach the authors should down sample the scifi 100k 200k to the same number of nuclei than the 16k libraries (about 4k), then perform a common integration. Thereby you will be able to assess if the peak calling and data quality are similar or better/worst.

L87

Please clarify the use of the concept of cell barcode collisions. Usually barcode collisions occur when one barcode maps to multiple cells. But here you can separate the different nuclei by the tn5 barcodes. So, what represent collisions here?

For example, if you find both genotype (B73 and Mo17), it is good thing no, as you are able to deconvolute the signal with SNPs and tn5 barcodes? Does collision here refer to two genotypes with having the same cell barcode and tn5 barcode? Then in your 8-genotype assay it shouldn't happen, as each genotype have a different tn5 barcode.

Even in the M&M it is said " Cell barcode collisions were identified using a previously described approach [4]" but no mention of "collisions" in this publication. When you assess the number of nuclei per droplet between Fig S1d and f and S1h there is a big discrepancy in the median rate. Can you explain?

Also I am not sure Fig S1h is cited in the manuscript.

Minor comments:

L73

Could you clarify how both genotypes are mixed?

Did you extract the nuclei separately and then mixed an equal amount of both. Or did you put all the seedling together and performed a single nuclei extraction?

L227

please add the approximated number of reads per nuclei you obtained. This parameter would be important for user to know how much they should sequence. Also, you stated using a Novaseq S4 chip run. Could you state how many and how much of each library you loaded?

ie: lib 16k 15% + lib 100k 25% + lib 200k 60% How many nuclei sequenced and how many reads per nuclei?

Several graphs are in Log10 which is difficult to read. It could stay a log scale.

e.g., could you change Fig 1e and S1e,g instead of 3 put 1000 of 1k etc.? it is much easier to read, same for S4.

**Reviewer 2**

**Are you able to assess all statistics in the manuscript, including the appropriateness of statistical tests used?**Yes, and I have assessed the statistics in my report.

**Comments to author:**

In this manuscript, Zhang and co-authors describe a novel technique called single-cell combinatorial fluidic indexing ATAC-sequencing ("scifi-ATAC-seq"), which aims to overcome the limitations of current single-cell methods in terms of throughput and cost. The utilization of scifi-ATAC-seq holds promise in the field of single-cell genomics, particularly in the identification of candidate cis-regulatory elements at genome-wide resolution with cell-type specificity. By combining the advantages of barcoded Tn5 pre-indexing and droplet-based single-cell ATAC-seq, this technique enables the simultaneous profiling of many nuclei from multiple samples, thereby improving the efficiency and scalability of single-cell ATAC-seq experiments.

Here are some comments and suggestions for the manuscript improve:

1. The authors did not explicitly state the maximum number of cells loaded in scifi-ATAC-seq. Is 200k cells the maximum number of cells loaded in scifi-ATAC-seq? However, since nuclei are pre-indexed in a 96-well plate, the maximum number of cells per well would depend on the specific experimental setup and the capacity of each well. It is not possible to determine the exact maximum number of cells per well without additional information from the authors. Regarding barcode collision rate, the level of tolerance in scifi-ATAC-seq would depend on the specific experimental design and the number of unique barcodes used. The authors did not provide specific information about the level of barcode collision tolerated in their study.

2. In scifi-ATAC-seq, a large number of nuclei are overloaded to increase throughput. However, this can lead to uneven distribution of cells in the microfluidic system, resulting in significant differences in cell numbers across droplets. This may introduce bias and affect the reliability and consistency of the data. Did the authors consider adjusting cell nucleus concentration and loading flow rate to maintain an even distribution of cells in the microfluidic system and reduce variations in cell numbers? Or did they utilize statistical methods and correction techniques to address the bias introduced by cell overcrowding?

3. Due to the use of 96 different barcode combinations, pre-indexed nuclei may suffer from barcode cross-contamination, Did the authors compare the probabilities of barcode contamination for different numbers of cells?

4. The article only compares scATAC-seq and scifi-ATAC-seq methods without comparing them to other relevant methods.

The author should consider comparing the scifi-ATAC-seq method with other scATAC-seq methods in a more comprehensive manner to evaluate its advantages and disadvantages in terms of data quality and result consistency.

5. Although the article mentions the application of the method to maize seedlings, it lacks a detailed discussion on its applicability to other sample types and species. It is hoped that the authors can include a discussion on the broad applicability and limitations of the method.

6. Are there any differences in the number of single cells obtained from each sample, and is the sequencing throughput consistent? Using 8 samples for the single-cell multi-sample experiment may not necessarily represent the maximum sample size. The article should provide a clear justification for selecting this sample size and discuss whether it adequately captures the diversity and variability within the experimental context. Additionally, it is crucial to assess whether there are differences in the number of single cells obtained from each sample and if the sequencing throughput is consistent across samples.

**Reviewer 3**

**Are you able to assess all statistics in the manuscript, including the appropriateness of statistical tests used?**Yes, and I have assessed the statistics in my report.

**Comments to author:**

The manuscript "Massive-scale single-cell chromatin accessibility sequencing using combinatorial fluidic indexing" by Zhang et al. presented the innovative technique scifi-ATAC-seq for large scale single cell chromatin accessibility profiling. The technique cleverly combined the commercial scATAC platform by 10X Genomics with pre-indexing nuclei of different samples/genotypes by distinct Tn5 barcodes, thereby allowing more nuclei to be profiled per experiment and greatly reducing the costs. Through experiments combining diverse maize genotypes, the authors demonstrated that compared to the standard 10X scATAC-seq protocol, scifi-ATAC-seq achieved similar data quality while the increased throughput enabled better interrogation of rare cell types. The manuscript was well written, the data analysis was thorough and clearly presented, and the technique and protocol will be of broad interest for the genomics community.

I have only three minor questions and suggestions:

1. The genotype identification of the nuclei was described as "SNP counts" or "Proportion of SNPs". Examples: Fig. 1 (g-i) legend: "Scatterplot displaying SNP counts per cell for B73 and Mo17, color-coded by genotype classification." Fig. 2(b) legend: "Distribution of proportion of SNPs in target genotypes". I guess the authors were referring to the number of reads or proportion of reads mapped to the B73, Mo17 or the target/non-target genomes. Please clarify. Also, it is not clear to me what the x-axis of Fig 2(b) "Total Tn5 number (log10)" is.

2. Fig. 2 and legend refers to "target genotypes" but this term was not defined in the main text. The method refers to "expected genotypes" were assigned by matching known Tn5 barcodes. Please clarify in the main text.

3. In the 7-genotype-mixed scifi-ATAC-seq experiment, can the author present a quantification of the reproducibility between B73 rep1 and rep2? This would help assess the reproducibility of the technique. Also, why did B73 rep1 have much larger number of nuclei compared to rep2 (and other genotypes?)

**Authors Response**

**Point-by-point responses to the reviewers’ comments:**

Reviewer #1: ===

*This manuscript describes very clearly an elegant high throughput method for single nuclei ATAC-seq. While the method was already published in animals, this represents an important novelty in the field of plant science with numerous applications and a broad audience.*

*The manuscript is well written, clearly presented, despites a few improvements that can be made, and results support the conclusions.*

R: We thank the reviewer for their positive evaluation of our work and their constructive comments to improve our study.

*Major comments:*

*While this is a short report, more insights on the underlying biology would really improve the quality and value of the manuscript.*

*One can imagine that a more thorough biological analysis of the generated data will follow this paper in the future, but more attention to the biological relevance of the data produced is needed. For example, did the authors find that maize genetic divergence of the different genotypes is correlated with more differential ACR etc.*

*Or, for example, does the tropical variety have more or less ACR in specific types of genes?*

*Is there any GO term enrichment for the differential ACR across genotypes and then which cell type seems to be the most divergent between genotypes etc?*

R: We thank the reviewer for these comments and suggestions. We agree that the data generated in this paper is a valuable resource for the community and that a thorough biological analysis would reveal interesting results. However, the goal of this manuscript is to communicate the development of an innovative method for profiling chromatin accessibility across hundreds of thousands of single cells from a single reaction. We will make all the raw and processed data openly accessible to the public if other researchers wish to pursue the biological relevance of these samples.

However, the reviewer makes a good point about ACRs and genetic distance. Our analysis supports that there is such a correlation revealing the data to be of high quality. We have amended the text as follows to describe this result(L149-152).

“To further validate the biological relevance and quality of the data, we conducted a Spearman correlation analysis with chromatin accessibility among the eight libraries (Fig 2c). Our findings reveal that the clustering of different genotypes aligns well with the known genetic relationships in maize, notably grouping all genotypes from non-stiff stalk (NSS) together. “

We also examined genotype-specific Accessible Chromatin Regions (gtACRs) and their closest genes. The number of gtACRs for each genotype ranged from 1,918 to 3,015, with tropical varieties Tzi8 and the mixed tropical-temperate line Mo18W showing a higher number of gtACRs among the seven genotypes (Fig 2i). For Tzi8, the gtACR-associated genes were most enriched in photomorphogenesis (p-value < 1e-4) and regulation of response to red or far-red light (p-value < 1e-3). These findings may reflect its distinct adaptation response to day length or light intensity. The full gene list and enrichment result were provided in Supplementary Table 8 and Supplementary Table 9.

We have incorporated this information into the manuscript to reflect these findings(L164-167).

“For tropical line Tzi8, the gtACR-associated genes were most enriched in photomorphogenesis (p-value < 1e-4) and regulation of response to red or far-red light (p-value < 1e-3). These findings may reflect its distinct adaptation response to day length or light intensity (Table S8,9).”

*Could the authors also comment on the minimum number of SNPs per genotype to be used here. As typically each read is about R1:49bp and R2 50bp, could you state how many SNPs per kb are necessary to be certain to correctly assign reads to a specific genotype. Maybe add in the manuscript the SNP rate between genotypes.*

R: We agree with the reviewer that cell genotyping is an important part of the workflow that would benefit from further clarification. In our approach, we are not assigning reads to genotypes but rather assigning cells to specific genotypes. Initially, we identify cells based on the cell barcode and subsequently use all the reads within each cell to assign a genotype. In this process, we set a minimum threshold of 50 reads covering common variants within a cell to confidently assign the genotype based on the alleles present. The total SNP number between genotypes is approximately 1.6M and SNP rate is about 0.70/kb (see figure below).

To make this clearer we updated the description of methods as below(L301-303):

“A minimum threshold of 50 reads covering common variants within a cell was used to confidently assign the genotype. And the total SNP number between genotypes is around 1.6 million and the SNP rate is about 0.70/kb.”

*Fig. 1, k,i The comparison of data quality between 16k and scifi 100k 200k is biased due to the number of nuclei. It is quite expected to have a better coverage and cell calling with 10 to 20x more nuclei.*

*Moreover, in L120-121 the resolution of ACR can be difficult to discern as the 100-200k cells are driving the integration and 16k libraries that could have a different batch effect and their clustering can be affected by the common integration. An integration of only the 16k libraires should help the comparison. Do you find the same cell type with only the 16k nuclei, or do you need more nuclei in general?*

*To perform a more rigorous approach the authors should down sample the scifi 100k 200k to the same number of nuclei than the 16k libraries (about 4k), then perform a common integration. Thereby you will be able to assess if the peak calling and data quality are similar or better/worst.*

R: We are grateful for the comment and suggestions. Indeed, increasing the number of nuclei improves cell identification and coverage, which benefits peak calling and downstream analysis at the cell-type level. This was a major goal of our study.

To address genotype and batch effects, we used the R package Harmony with non-default parameters (do_pca=F, vars_use=c("library", "genotype"), tau=c(5), lambda=c(0.1, 0.1), nclust=50, max.iter.cluster=100, max.iter.harmony=30). We interpreted the integration as being successful given our observation of no significant bias in cell-type proportions between the two technologies (Fig S2d. Spearman correlation: 0.62, p-value < 0.05). From a biological standpoint,the marker accessibility patterns among clusters were similar when we split the embedding by the different libraries/technologies, such as phloem/procambium marker gene ZmSMXL3 (Fig 1l). Our analysis suggests that there are no substantial batch effects that could significantly impact cell clustering and annotation.

To compare the nuclei quality between scifi-ATAC-seq and scATAC-seq, we randomly selected 100 cells for each cell type or library and checked the distribution of the number of Tn5 integrations for each cell type. We observed that scifi-ATAC-seq has a lower median number of Tn5 integrations (Figure S4a,b). This difference may be attributed to the relatively low sequencing depth of scifi-ATAC-seq (7.6k reads per cell) compared to scATAC-seq (51.7k reads per cell).

There is potential to identify more rare cell types or cell states with an increased number of Tn5 integrations or nuclei numbers. To test whether the nuclei number or Tn5 integration number could affect the cell clustering, we clustered the nuclei from scifi-ATAC-seq, subsampled scifi-ATAC-seq nuclei to an equal number with scATAC-seq, and nuclei from scATAC-seq separately with the same parameters. There were 9 clusters for the subsampled scifi-ATAC-seq and scATAC-seq, while the cluster number increased to 16 in the whole scifi-ATAC-seq dataset. These analyses suggest that the lower Tn5 integration in scifi-ATAC-seq might not significantly affect clustering in this study, while having more nuclei in scifi-ATAC-seq could potentially decipher the cell heterogeneity at a higher resolution (Figure S4c-e).

The figures and manuscript have been updated accordingly(L116-122).

“To compare the nuclei quality between scifi-ATAC-seq and scATAC-seq, we randomly selected 100 cells for each cell type or library and checked the distribution of the number of Tn5 integrations for each cell type. We observed that scifi-ATAC-seq has a lower median number of Tn5 integrations (Figure S4a,b). While the lower Tn5 integration in scifi-ATAC-seq does not significantly affect clustering in this study, and the increased number of nuclei from scifi-ATAC-seq helps decipher cell heterogeneity (Figure S4c-e).”

*L87*

*Please clarify the use of the concept of cell barcode collisions. Usually barcode collisions occur when one barcode maps to multiple cells. But here you can separate the different nuclei by the tn5 barcodes. So, what represent collisions here?*

*For example, if you find both genotype (B73 and Mo17), it is good thing no, as you are able to deconvolute the signal with SNPs and tn5 barcodes? Does collision here refer to two genotypes with having the same cell barcode and tn5 barcode? Then in your 8-genotype assay it shouldn't happen, as each genotype have a different tn5 barcode.*

R: We agree it is important to clarify the use of ‘collision’ in this manuscript. In the context of this discussion, the term 'cell barcode' refers to the barcode used to determine cell identity. In the standard scATAC-seq, the cell barcode corresponds exclusively to the barcode derived from hydrolyzed GEM beads following microfluidic partitioning. However, in scifi-ATAC-seq, the cell barcode encompasses both the beads barcode and the Tn5 barcode. Cell barcode collision occurs when more than one cell shares the same cell barcode. In traditional droplet-based assays, cell barcode collision occurs if multiple cells enter one droplet. In scifi-ATAC-seq, cell barcode collision happens when multiple cells occupy the same droplet, and simultaneously, they share identical tn5 barcodes.

For the eight-genotype assay, we agree with the reviewer that all the cells represented by gray dots should not be attributed to barcode collision, but rather indicative of cells with a high index hopping contamination.

We have carefully revised the manuscript and corresponding figures to accurately reflect this interpretation(L308-315). Please see below:

“For the seven-genotype-mixed scifi-ATAC-seq data, expected genotypes were assigned by matching known Tn5 barcodes. Any reads that did not match the expected genotype were considered as belonging to another genotype. The same genotype calling approach described above was then used to assign nuclei to their expected genotype, identify mixtures of genotypes resulting from index hopping contamination. Given the varying cell numbers per well, we used a modeling-based approach to estimate the barcode collision rate by calculating the probability of obtaining any two cells from the same well in a four-nuclei droplet (mean nuclei number per droplet is 3.4, Fig S6c). “

Even in the M&M it is said " Cell barcode collisions were identified using a previously described approach [4]" but no mention of "collisions" in this publication. When you assess the number of nuclei per droplet between Fig S1d and f and S1h there is a big discrepancy in the median rate. Can you explain?

*Also I am not sure Fig S1h is cited in the manuscript.*

R: Thank you for reviewing the figures. In Fig S1h, the plot was initially based on nuclei type (non-collision vs. collision) and their nuclei number in the droplet. As there can be multiple nuclei in one droplet, the nuclei number is duplicated in the figure, which we agree could result in confusion. To address this, we have re-plotted the figure. Initially, we classified droplets as non-collision or collision based on the presence of any collision nuclei. Then, we plotted the nuclei number distribution based on droplet type. This adjustment ensures consistency with Fig S1d and Fig S1f.

Additionally, we identified an error in Fig S1d and Fig S1f. The original figures were generated from non-collision nuclei, but all nuclei should be used for this analysis. We have rectified this mistake in the updated plot. We can confirm that the original version of a similar figure in Fig S6c for the eight-library scifi-ATAC-seq assay was not affected by this mistake.

We revised the manuscript and cited the Fig S1h and further clarified the definition of cell barcode collisions in the “Methods” section as below(L284-290).

“The term 'cell barcode' refers to the barcode used to determine cell identity. In the standard scATAC-seq, the cell barcode corresponds exclusively to the barcode derived from hydrolyzed GEM beads following microfluidic partitioning. However, in scifi-ATAC-seq, the cell barcode encompasses both the beads barcode and the Tn5 barcode. Cell barcode collision occurs when more than one cell shares the same cell barcode. In traditional droplet-based assays, cell barcode collision occurs if multiple cells enter one droplet. In scifi-ATAC-seq, cell barcode collision happens when multiple cells occupy the same droplet, and simultaneously, they share identical Tn5 barcodes.”

*Minor comments:*

*L73*

*Could you clarify how both genotypes are mixed?*

*Did you extract the nuclei separately and then mixed an equal amount of both. Or did you put all the seedling together and performed a single nuclei extraction?*

R: We agree that the original text could result in confusion. Nuclei were isolated separately and then mixed at equal concentrations. We rephrased this in the Methods section to make this clearer(L220-222).

“Finally, after isolated nuclei for both genotypes separately, equal nuclei number of B73 and Mo17 were mixed together, and the nuclei density was adjusted to 0.5k~1k/μL with TAPS buffer.”

*L227*

*please add the approximated number of reads per nuclei you obtained. This parameter would be important for user to know how much they should sequence. Also, you stated using a Novaseq S4 chip run. Could you state how many and how much of each library you loaded?*

*ie: lib 16k 15% + lib 100k 25% + lib 200k 60% How many nuclei sequenced and how many reads per nuclei?*

R:. We agree that stating these parameters will be useful for readers. The libraries were sequenced in multiple batches, so it is hard to give an exact proportion for each library in the pools. However, we do have precise numbers of per cell read coverage that can inform future experiments.

The libraries were sequenced to an average depth of 7,617 read pairs per cell for scifi-ATAC-seq libraries with an average unique reads rate at 62.3%. And the scATAC-seq libraries were sequenced about 51.7k reads pairs per cell [4]. We have added this information in the method in “Library preparation and sequencing”(L247-249).

“The libraries were sequenced to an average depth of 7,617 read pairs per cell, with an average unique reads rate at 62.3%. The scATAC-seq libraries were sequenced about 51.7k reads pairs per cell [4].”

*Several graphs are in Log10 which is difficult to read. It could stay a log scale.*

*e.g., could you change Fig 1e and S1e,g instead of 3 put 1000 of 1k etc.? it is much easier to read, same for S4.*

R: Thanks for the suggestion and we have updated all related figures.

Reviewer #2: ===

*In this manuscript, Zhang and co-authors describe a novel technique called single-cell combinatorial fluidic indexing ATAC-sequencing ("scifi-ATAC-seq"), which aims to overcome the limitations of current single-cell methods in terms of throughput and cost. The utilization of scifi-ATAC-seq holds promise in the field of single-cell genomics, particularly in the identification of candidate cis-regulatory elements at genome-wide resolution with cell-type specificity. By combining the advantages of barcoded Tn5 pre-indexing and droplet-based single-cell ATAC-seq, this technique enables the simultaneous profiling of many nuclei from multiple samples, thereby improving the efficiency and scalability of single-cell ATAC-seq experiments.*

R: We thank the reviewer for their positive evaluation of our work and constructive suggestions.

*Here are some comments and suggestions for the manuscript improve:*

*1. The authors did not explicitly state the maximum number of cells loaded in scifi-ATAC-seq. Is 200k cells the maximum number of cells loaded in scifi-ATAC-seq? However, since nuclei are pre-indexed in a 96-well plate, the maximum number of cells per well would depend on the specific experimental setup and the capacity of each well. It is not possible to determine the exact maximum number of cells per well without additional information from the authors. Regarding barcode collision rate, the level of tolerance in scifi-ATAC-seq would depend on the specific experimental design and the number of unique barcodes used. The authors did not provide specific information about the level of barcode collision tolerated in their study.*

R: We are grateful for the reviewer's insightful comment. The maximum number of nuclei is determined by the number of unique Tn5 barcodes. In our experiment, we used 96 Tn5 barcodes, and the barcode collision rate was about 9.52% which is acceptable for 200k input. Moreover, we have the flexibility to easily scale up the barcode indices to accommodate a higher number of nuclei, which will decrease the barcode collision rate. We have added a discussion about the maximum number of nuclei for the scifi-ATAC-seq with 96 Tn5 barcodes in the “Supplementary Note” at the end of Supplementary methods. Please find the detail in the supplementary note or our response for your #5 comment.

Barcode collisions happen in every single cell method and can be typically removed using an array of doublet detection tools, such as scDblfinder, AMULET, ArchR [16-19]. For 10X scATAC-seq, a suggested barcode collision rate of approximately 8.0% has been reported for 16k input nuclei. The acceptable level of barcode collision depends on the experimental design and the goals of the analysis. In Cusanovich's study, they successfully discriminated chromatin states between different cell lines and within cell lines with a collision rate of 11% [10].

*2. In scifi-ATAC-seq, a large number of nuclei are overloaded to increase throughput. However, this can lead to uneven distribution of cells in the microfluidic system, resulting in significant differences in cell numbers across droplets. This may introduce bias and affect the reliability and consistency of the data. Did the authors consider adjusting cell nucleus concentration and loading flow rate to maintain an even distribution of cells in the microfluidic system and reduce variations in cell numbers? Or did they utilize statistical methods and correction techniques to address the bias introduced by cell overcrowding?*

R: Thanks for the suggestion. We agree that the uneven distribution of cells in the microfluidic system can be a technical issue that affects the data quality, especially cell barcode collision and index hopping contamination within the droplets containing numerous cells. But in scifi-ATAC-seq, we could easily identify the droplets with overcrowded nuclei by checking the nuclei number in each droplet. And by combining a barcoded Tn5 pre-indexing step, we are further able to decrease the barcode collision rate and index hopping contamination. Further comparison of nuclei quality for droplets containing 1 to 10 nuclei revealed no significant bias in several quality metrics, including the proportion of reads around the transcription start site (TSS), fraction of reads in peaks (FRiP), and unique Tn5 insertions per cell (Fig. S2a-c).

The total nuclei volume and the loading flow rate were fixed in 10X single cell manual and equipment, providing increased stability and repeatability. scifi-ATAC-seq offers the flexibility to easily scale up Tn5 barcode indexes to further decrease the barcode collision and index hopping contamination.

We have incorporated this information into the manuscript and updated Fig S2a-c to reflect these findings(L99-101).

“For the droplets containing 1 to 10 nuclei, there is no obvious bias for several quality metrics, such as the proportion of reads around the TSS, FRiP score, unique Tn5 insertions per cell (Fig. S2a-c)”

*3. Due to the use of 96 different barcode combinations, pre-indexed nuclei may suffer from barcode cross-contamination, Did the authors compare the probabilities of barcode contamination for different numbers of cells?*

R: Thank you for the suggestion. We conducted a comparison of the barcode contamination rate in droplets with varying numbers of cells in both the 100k and 200k input scifi-ATAC-seq experiments. There is a noticeable increase in the contamination rate with more cells in the droplet, but it remains at a low level (<5%).

We have incorporated this information into the manuscript (L97-103) and updated Fig S2d to reflect these findings.

“As expected, the total number of the nuclei in the droplet with barcode collisions and the number of Tn5 insertions in nuclei with barcode collisions is significantly higher than non-collision droplets or nuclei (see Fig. S1d-h, q-value < 10e-16). For the droplets containing 1 to 10 nuclei, there is no obvious bias for several quality metrics, such as the proportion of reads around the TSS, FRiP score, unique Tn5 insertions per cell (Fig. S2a-c) whereas there is a noticeable increase in the contamination rate with more cells in the droplet, but it remains at a low level (<5%) even in the droplets with 10 nuclei (Fig S2d).”

*4. The article only compares scATAC-seq and scifi-ATAC-seq methods without comparing them to other relevant methods.*

*The author should consider comparing the scifi-ATAC-seq method with other scATAC-seq methods in a more comprehensive manner to evaluate its advantages and disadvantages in terms of data quality and result consistency.*

R: We appreciate the comment. Our goal is to show that the scifi-ATAC-seq can dramatically increase the throughput of the 10X Genomics platform, which is most commonly used for scATAC-seq data generation. Comparing the ATAC-seq data from the 10X Genomics platform and other (non 10X Genomic) platforms is not the goal of this study. Most importantly, there is no publicly available maize leaf single-cell ATAC-seq data from these other methods to allow us to make this comparison. Otherwise, we agree that additional comparison would be useful to the readership.

*5. Although the article mentions the application of the method to maize seedlings, it lacks a detailed discussion on its applicability to other sample types and species. It is hoped that the authors can include a discussion on the broad applicability and limitations of the method.*

R: We agree with the reviewer that a discussion of the applicability of our approach to other models would be useful. Due to the space limitation for the short report, we added this to the “Supplementary Note” at the end of Supplementary methods.

“Supplementary Note:

1. Application of scifi-ATAC-seq for different species and tissues:

In theory, there should be no sample or species limits for scifi-ATAC-seq, provided that high-quality nuclei can be prepared. We have successfully obtained high-quality nuclei for numerous plant species with the current nuclei isolation protocol, including maize, soybean, rice, wheat, barley, Arabidopsis, and poplar, across various tissue types such as leaf, root, nodule, hypocotyl, and developing seed. We recommend validating this nuclei isolation protocol by assessing nuclei quality under a microscope and evaluating the data quality through regular/non-single-cell ATAC-seq prepared from the isolated nuclei.

2. Maximum samples in one scifi-ATAC-seq assay:

In theory, it is feasible to mix up to 96 samples in one assay, given the use of 96 barcodes in this study. However, in practice, there is a risk of nuclei degradation if they remain in the buffer for an extended period. The nuclei isolation for each sample takes approximately 4 minutes, and an additional 30 minutes are needed for nuclei purification for all samples. In the 8-sample scifi-ATAC-seq, we aim to complete the entire nuclei preparation within 1 hour to minimize the risk of nuclei degradation during the process. Including more samples is possible if a sufficient number of high-quality nuclei can be obtained in the given time frame. From the 8-sample scifi-ATAC-seq with a 300k input, we obtained about 120k nuclei following strict QC. Therefore, it should be feasible to include up to 12 samples in the 300k input scifi-ATAC-seq assay, ensuring an average of 10k nuclei per sample, which is generally acceptable for most experiments. In animals, a similar approach called txci-ATAC-seq has been developed. It provides a protocol for preparing scATAC-seq with frozen nuclei, offering the potential to profile up to 96 samples simultaneously [34].

3. Maximum numbers of nuclei in one scifi-ATAC-seq assay:

The maximum number of nuclei is primarily determined by the number of unique barcodes, with more indexes reducing the probability of barcode collision when multiple nuclei are present in one droplet. In other words, in the 96-barcodes experiment, the probability of barcode collision in the droplet with a certain number of nuclei is expected to be similar, regardless of the total number of loaded nuclei. To verify this, we compared barcode collision rates for droplets containing one to twenty nuclei in both 100k and 200k input scifiATAC-seq. The results showed an increase in the barcode collision rate with more nuclei in the droplet, yet showing similarity between 100k and 200k input scifi-ATAC-seq, where the collision rate is approximately 15% for droplets with 10 nuclei (Fig S9b).

However, the distribution of the number of nuclei in droplets for 100k, 200k, and 300k input nuclei is not a normal distribution but a biased distribution, with most droplets containing one to ten nuclei (Fig S1d,f; Fig S6c). Therefore, it is possible to further decrease the barcode collision rate by removing droplets with a high number of nuclei. For instance, the barcode collision rate could be reduced to about 4% and 6% when filtering droplets with more than 10 nuclei while retaining over 90% and 80% of the total non-collision nuclei for 100k and 200k input scifi-ATAC-seq, respectively (Fig S9c-e).

In one assay from the 10X Genomics Chromium Controller, approximately 70k droplets were generated, and about 90% of these droplets could be filled with 383k input nuclei (Fig S9a). However, as the number of input nuclei increases, there is a higher risk of chip clogging due to the high density of nuclei. We believe it might be feasible to load 400k nuclei in each run and selectively retain only the droplets with no more than 10 nuclei for downstream analysis using the 96-barcodes scifi-ATAC-seq method.

4. Potential limits for mixing samples from different species in one assay:

There is a potential for sequencing throughput bias among nuclei from different species if they contain significantly different sizes of chromatin accessibility regions due to variations in genome size and as well as GC content variation among species.”

*6. Are there any differences in the number of single cells obtained from each sample, and is the sequencing throughput consistent? Using 8 samples for the single-cell multi-sample experiment may not necessarily represent the maximum sample size. The article should provide a clear justification for selecting this sample size and discuss whether it adequately captures the diversity and variability within the experimental context. Additionally, it is crucial to assess whether there are differences in the number of single cells obtained from each sample and if the sequencing throughput is consistent across samples.*

R: We are grateful for the reviewer’s comment. On average, we obtained 15,582 QC-filtered nuclei for each sample, approximately twice the yield of a regular 16K input scATAC-seq. We observed a higher proportion of nuclei for B73 rep1. While we are uncertain about the exact reason for this observation, it could potentially be attributed to standard error of measuring nuclei concentration. However, we are pleased to note that despite this discrepancy, the levels of estimated barcode collision (7.6%) and index hopping contamination (1.88%) remain at low levels. This suggests that scifi-ATAC-seq is robust and can tolerate such technical variation (Fig S4d).

In theory, the sequencing throughput should be similar for each nucleus, as all the samples are from the same species and tissue type, which means they are likely to have a similar number of accessible chromatin regions. We checked the correlation between sequencing throughput and nuclei number for all samples and the spearman correlation is very high at 0.98 (Fig. S6d). However, we agree with the reviewer that this could pose a limitation for scifi-ATAC-seq when mixing samples from different species with significantly different amounts of accessible chromatin regions within one assay.

We have updated the new finding in the updated manuscript(L145-147):

“As expected, there was high correlation between sequencing throughput and nuclei number for all samples (R=0.98, Fig. S6d).”

The primary goal of scifi-ATAC-seq using the eight mixed samples is to assess the robustness of profiling single-cell chromatin accessibility across multiple samples within a single reaction. We are not intending to test the maximum sample size that can be included in scifi-ATAC-seq. Given that it takes approximately 4 minutes to isolate nuclei for one sample and about 30 minutes for nuclei purification, our aim is to complete the entire nuclei preparation within about 1 hour to prevent nuclei degradation during the process. It is feasible to include more samples for this experiment as long as we obtain enough high-quality nuclei for the analysis.

We added these discussions in the “Supplementary Note”, please see previous comment for detail.

Reviewer #3: ===

*The manuscript "Massive-scale single-cell chromatin accessibility sequencing using combinatorial fluidic indexing" by Zhang et al. presented the innovative technique scifi-ATAC-seq for large scale single cell chromatin accessibility profiling. The technique cleverly combined the commercial scATAC platform by 10X Genomics with pre-indexing nuclei of different samples/genotypes by distinct Tn5 barcodes, thereby allowing more nuclei to be profiled per experiment and greatly reducing the costs. Through experiments combining diverse maize genotypes, the authors demonstrated that compared to the standard 10X scATAC-seq protocol, scifi-ATAC-seq achieved similar data quality while the increased throughput enabled better interrogation of rare cell types. The manuscript was well written, the data analysis was thorough and clearly presented, and the technique and protocol will be of broad interest for the genomics community.*

R: We thank the reviewer for their positive evaluation of our work and for their suggestions to improve the study.

*I have only three minor questions and suggestions:*

*1. The genotype identification of the nuclei was described as "SNP counts" or "Proportion of SNPs". Examples: Fig. 1 (g-i) legend: "Scatterplot displaying SNP counts per cell for B73 and Mo17, color-coded by genotype classification." Fig. 2(b) legend: "Distribution of proportion of SNPs in target genotypes". I guess the authors were referring to the number of reads or proportion of reads mapped to the B73, Mo17 or the target/non-target genomes. Please clarify. Also, it is not clear to me what the x-axis of Fig 2(b) "Total Tn5 number (log10)" is.*

R: We agree with the reviewer “the number of B73/Mo17 reads” is more accurate for Fig. 1g-i. And we have revised related text.

For Fig. 2(b) and related figures (Fig. S6e-l), we renamed the axis as “Proportion of variant-covering reads in expected genotype”. And the "Total Tn5 number (log10)" is the total number of Tn5 integration in nuclei. And we have renamed the x-axis as “Total number of Tn5 integration”.

*2. Fig. 2 and legend refers to "target genotypes" but this term was not defined in the main text. The method refers to "expected genotypes" were assigned by matching known Tn5 barcodes. Please clarify in the main text.*

R: We appreciate the comment. We have corrected all instances of “target genotypes” to “expected genotypes”. And further clarified in main text(L143-145).

“We assigned all nuclei with the expected genotypes by matching known Tn5 barcodes and identified 133,524 clean nuclei.”

*3. In the 7-genotype-mixed scifi-ATAC-seq experiment, can the author present a quantification of the reproducibility between B73 rep1 and rep2? This would help assess the reproducibility of the technique. Also, why did B73 rep1 have much larger number of nuclei compared to rep2 (and other genotypes?)*

R: Thanks for the suggestion.

We assessed the reproducibility between B73 rep1 and rep2 by calculating the Spearman correlation coefficient based on the chromatin accessibility of all accessible regions across samples (Fig 2c), and the correlation was remarkably high at 0.97.

While we are not precisely certain for the larger number of nuclei in B73 rep1, we suspect it is likely due to technical variation stemming from the measurement of nuclei concentration. Using an automatic cell counter for nuclei counting might offer more stability in this regard. Despite this discrepancy, we are pleased to observe that the levels of estimated barcode collision (7.6%) and index hopping contamination (1.88%) remain low and within an acceptable range.

**Second round of review**

**Reviewer 1**

The authors have thoroughly addressed my comments.

**Reviewer 2**

The author almost addressed my concerns.
